# EGFR Signaling: Friend or Foe for Cartilage?

**DOI:** 10.1002/jbm4.10177

**Published:** 2019-02-13

**Authors:** Ling Qin, Frank Beier

**Affiliations:** ^1^ Department of Orthopaedic Surgery Perelman School of Medicine University of Pennsylvania Philadelphia PA USA; ^2^ Department of Physiology and Pharmacology University of Western Ontario London Canada; ^3^ Western Bone and Joint Institute University of Western Ontario London Canada

**Keywords:** EPIDERMAL GROWTH FACTOR RECEPTOR (EGFR), OSTEOARTHRITIS, CARTILAGE

## Abstract

Recent studies using genetically modified mice, pharmacological approaches, and human samples have highlighted an important role for the epidermal growth factor receptor (EGFR), selected ligands, and downstream components in endochondral bone formation and joint homeostasis. Although most data demonstrate an important function of this pathway in endochondral ossification and articular cartilage growth, conflicting results on its role in osteoarthritis have been reported. In some contexts, inactivation of EGFR signaling has been shown to protect joints from surgically induced osteoarthritis, whereas in others, similar manipulations worsened joint pathology. The current review summarizes recent studies of cartilage EGFR signaling in long bone development and diseases, provides potential explanations for the reported discrepancies, and suggests directions for future work to clarify the potential of this pathway as target for osteoarthritis treatment. © 2019 The Authors. *JBMR Plus* published by Wiley Periodicals, Inc. on behalf of American Society for Bone and Mineral Research.

## Introduction

The epidermal growth factor receptor (EGFR) represents one of the longest‐studied growth factor receptors in research history. Its prototype ligand, epidermal growth factor (EGF), was first discovered by Dr Stanley Cohen in crude extracts of mouse salivary gland more than half a century ago. Those extracts had potent actions on precocious tooth eruption and eyelid opening in newborn mice.^1^ Twenty years later, Dr Cohen purified its receptor, the 170 kD EGFR, from human A‐431 cells^2^, ^3^ and demonstrated that it is a receptor tyrosine kinase (RTK).^4^ Because of his seminal work in the growth factor signaling field, Dr Cohen was awarded the Nobel Prize for Medicine and Physiology in 1986.

**Figure 1:**
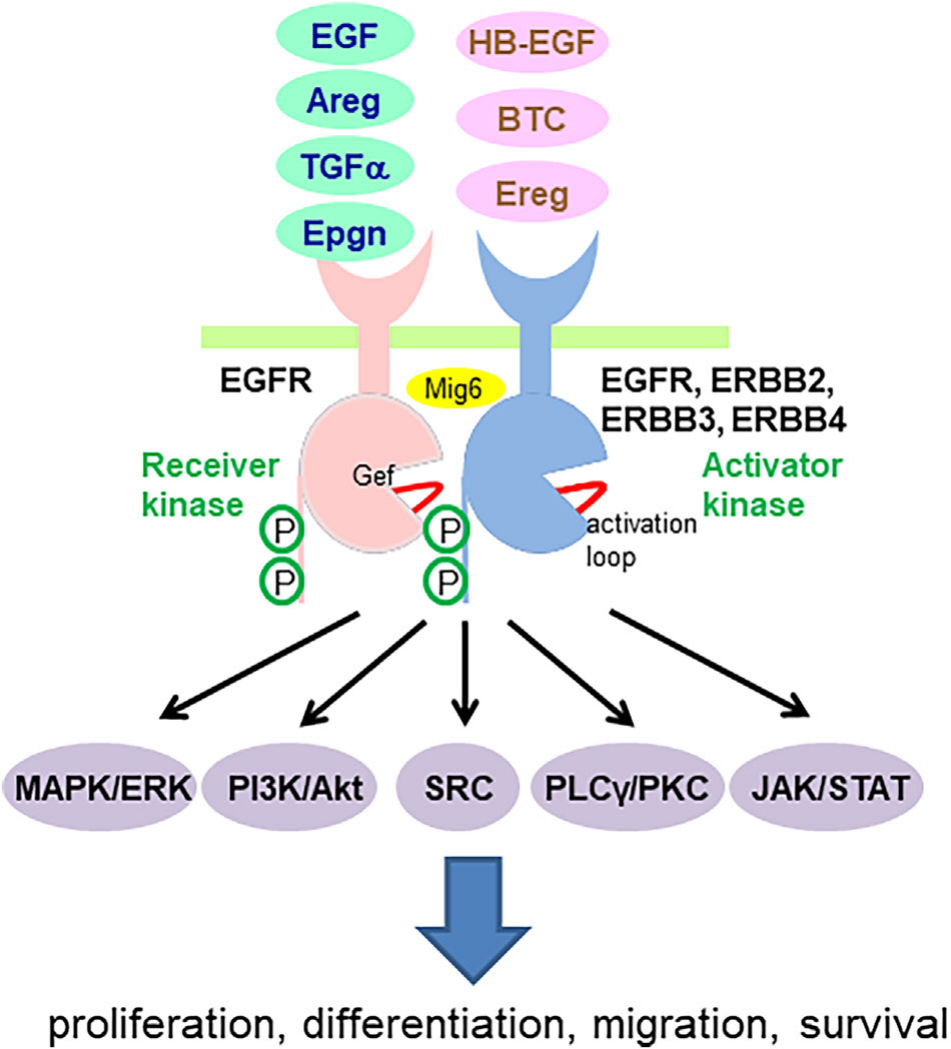
Ligand‐induced EGFR homo‐ and heterodimerization and their downstream signal transduction pathways. EGFR ligands include EGF, amphiregulin, TGFα, and Epgn (green), which only bind to EGFR, and HB‐EGF, BTC, and Ereg (pink), which bind to both EGFR and ERBB4. Binding of EGFR ligand promotes asymmetric dimer formation between EGFR and another ligand‐bound EGFR family member (EGFR, ERBB2, ERBB3, or ERBB4), in which part of one intracellular tyrosine kinase domain (TKD), the Activator, makes intimate contacts with another part of the second TKD, the Receiver, in a head‐to‐tail fashion. This asymmetric dimer interface can be disrupted by binding of Mig6, a negative feedback inhibitor of EGFR. The TKD contains an activation loop next to an ATP binding site. EGFR inhibitors, such as Gefitinib, AG1478, and Erlotinib, bind to this site and thus prevent ATP docking. This unique asymmetric dimerization of receptors activates the receiver kinases and results in trans‐autophosphorylation of receptors. Activation of EGFR induces multiple downstream signaling pathways as well as various biological outcomes.

The complexity of the EGFR system grows phylogenetically in the animal kingdom. C. elegans has a single receptor and a single ligand,^5^ while Drosophila possesses one receptor and four ligands.^6^ Rodents, like humans, have four receptors (EGFR/ERBB1/HER2, ERBB2/HER2/Neu, ERBB3/HER3, and ERBB4/HER4) and 11 ligands, including EGF, transforming growth factor α (TGFα), amphiregulin (Areg), heparin‐binding EGF (HB‐EGF), betacellulin (BTC), epiregulin (Ereg), epigen (Epgn), and neuregulins 1‐4^7^ (Fig. 1). All EGFR family members function as either homo‐ or heterodimers. Compared with EGFR, ERBB2 does not contain a ligand‐binding domain and ERBB3 does not contain a kinase domain. EGFR is the only receptor for EGF, TGFα, Areg, and Epgn and can also transduce signals for other ligands except neuregulins. All EGF‐like ligands are first synthesized as a membrane protein. Thus, they require sheddases such as ADAM10 and 17 for ectodomain shedding for release from the expressing cells to act on target cells for autocrine or paracrine stimulation of EGFR signaling.^8^

In stark contrast to other RTKs, the activation of EGFR occurs through receptor‐mediated dimerization followed by trans‐autophosphorylation of the intracellular kinase domain. Specifically, each EGFR monomer binds an EGF ligand, exposes its unique dimerization arm, and then forms an asymmetric dimer.^9^ Within this dimer, the C‐terminus of one EGFR containing multiple phosphorylation sites inserts into the kinase domain of another EGFR, an allosteric interaction that activates the receiving kinases and results in trans‐autophosphylation.^10^ In the absence of ligands, EGFR can also be activated by other receptors, such as G‐protein‐coupled receptors and cytokine receptors, and therefore can act as signal integrator.^11^ Subsequently, phosphorylated EGFR initiates myriad key signaling pathways, among which the most studied are MAPK/ERK, PI3K/Akt, SRC, PLCγ/PKC, and JAK/STAT. Because these signaling pathways are interlinked, EGFR activation actually stimulates an entire signaling network associated with a wide number of outcomes, such as cell proliferation, differentiation, migration, and survival.

Almost all cell types except hematopoietic cells express EGFR family members. Hence, it is no surprise that ERBBs are important for normal development and physiology of vertebrates. Indeed, knockout of any one of them in mice causes embryonic or perinatal lethality.^12^ Particularly, the death of EGFR null mice is owing to developmental abnormalities in brain, skin, lung, and gastrointestinal tract.^13^ A recurring theme of physiological EGFR function in multiple organs is its ability to maintain tissue‐specific stem cells and promote their self‐renewal. For example, EGFR signaling is important for epidermal homeostasis and hair follicle development, and modulation of EGFR signaling impacts the behavior of keratinocyte stem cells.^14^ Similarly, EGFR and its main ligand, EGF, constitute one of the most important growth factor signaling pathways with significant effects on the proliferation, survival, migration, and differentiation rate of embryonic and adult neural precursors.^15^ In small intestinal crypts, EGF and TGFα are among the major signals that maintain the population of Lgr5 stem cells.^16^ In bone, we have shown that activation of EGFR signaling, most likely by Areg after parathyroid hormone treatment, stimulates proliferation, survival, and migration of osteoprogenitors.^17^, ^18^ Therefore, EGFR function is closely related to the regulation of tissue‐specific stem cells and progenitors under physiological, injury, and disease conditions.

## Long Bone Development by Endochondral Ossification

In mammals, long bones are formed through a highly spatiotemporally regulated process termed endochondral ossification. Early in development, cells migrate to the future bone sites, exclude vessels, and form mesenchymal condensation. Within those cells, Sox9 expression is upregulated to induce chondrogenesis. When the resultant cartilage anlage grows to a certain size, its center cells stop proliferating and become enlarged, hypertrophic chondrocytes.^19^ Meanwhile, mesenchymal cells at the condensation boundary begin to flatten, elongate, and form the perichondrium, which upon stimulation by chondrocyte‐secreted Indian hedgehog (Ihh) develops into bone collar. Later, blood vessels initiated from perichondrium invade into the calcified cartilage matrix at the center of anlage, bringing osteoprogenitors with them. In conjunction with chondrocyte apoptosis and/or transdifferentiation of hypertrophic chondrocytes to osteoblasts, this leads to remodeling of cartilage tissue into metaphyseal trabecular bone and bone marrow to form the primary ossification center (POC).^19^, ^20^ Shortly after birth, the epiphyseal cartilage is excavated by canals invaginating from the periarticular region. These cartilage canals allow for the arrival of blood vessels and osteoprogenitors to the center of the epiphyseal cartilage to remodel the epiphyseal cartilage into subchondral trabecular bone and bone marrow, to form the secondary ossification center (SOC). The sequential development of the POC and SOC defines the location of the growth plate and articular cartilage, which both consist of chondrocytes. During this endochondral ossification process, the generation and dissolution of cartilage tissue are tightly regulated by multiple growth factors and hormones, among which EGFR signaling has been extensively studied in the past two decades.

## EGFR Signaling in Mesenchymal Condensation

Shortly after EGF had been found to be a potent mitogen for fibroblasts in the 1970s, its function was tested in many other cell types, including chondrocytes. Earlier studies have shown that EGF stimulates DNA synthesis and colony formation of chondrocytes but suppresses their differentiation into mature chondrocytes and blocks proteoglycan synthesis.^20^, ^21^, ^22^

During the early stage of limb development, the epithelial cells overlying the mesenchymal cells (apical ectodermal ridge, AER) direct mesenchymal condensation and chondrogenic differentiation. They form a signaling center that keeps the underlying mesenchymal cells in a proliferative and undifferentiated state. An elegant study using chick limb bud model revealed that TGFα and EGF are expressed in both AER and proliferating mesenchymal cells but not in cells that are undergoing chondrogenic differentiation and moving away from AER.^23^ In vitro limb mesoderm explant culture and micromass culture of mesenchymal cells demonstrated that activation of EGFR signaling stimulates limb outgrowth but inhibits differentiation of precursor cells into chondrocytes,^23^, ^24^ possibly through regulating downstream PKCα, ERK, and p38 MAPK pathways. Sox9 and Runx2 are two master transcription factors driving mesenchymal cells into chondrogenic lineage. It has been reported that TGFα suppresses these two factors in chondrocytes.^25^ Hence, similar to their action in other tissue‐specific stem cells and progenitors, EGF‐like ligands play an autocrine or paracrine role of maintaining the progenitor pool and preventing their premature differentiation into chondrocytes during the early mesenchymal stage of long bone development.

## EGFR Signaling in Growth Plate Development

The axial growth of long bones occurs through growth plates at their two ends. Located between SOC and POC, the growth plate tissue consists of chondrocytes arranged in a columnar and zonal manner. To achieve longitudinal growth, growth plate chondrocytes undergo resting, proliferation, differentiation, hypertrophy, and calcification stages, eventually encountering cell death to make way for bone formation. Hence, the overall bone growth is a combination of cartilage generation, which is determined by chondrocyte proliferation and hypertrophy, and cartilage degradation, which is determined by chondrocyte turnover and cartilage matrix removal.

**Figure 2:**
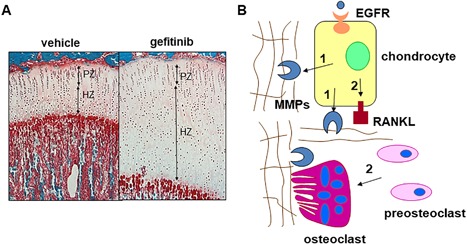
EGFR signaling is a critical pathway for normal growth plate development. (*A*) One‐month‐old rats received 7 days of Gefitinib treatment and their tibias exhibited a striking enlargement of the growth plate, particularly the hypertrophic zone. PZ = proliferative zone; HZ = hypertrophic zone. (*B*) A model of how EGFR signaling stimulates cartilage matrix degradation. Two mechanisms contribute to this function of EGFR. First, EGFR signaling upregulates the expression of MMPs (9, 13, and 14) in the growth plate and thus is responsible for cartilage extracellular matrix degradation. Second, EGFR signaling is important for RANKL expression in the growth plate and thus is responsible for osteoclastogenesis at the COJ. (Adapted from Zhang and colleagues.^29^)

In rodents with deficient EGFR activity, a common theme is delayed POC formation together with an expanded growth plate during early skeletal development (Fig. 2 *A*). At E15‐16 when the POC is first established in the wide‐type long bones, EGFR null mice^26^ or mice overexpressing Herstatin, a truncated soluble ErbB2 receptor that diminishes EGFR activity by impairing dimer formation among ErbB family members,^27^ still possess an uninterrupted hypertrophic zone at the POC site, with no vessel invasion and no cartilage matrix degradation. At birth, a few surviving EGFR null mice display elongated epiphyseal growth plates with a major expansion of the hypertrophic cartilage zone. To overcome the lethality problem, a knockin model was generated to replace the endogenous mouse Egfr by a human Egfr cDNA. These mice have low EGFR activity but can survive for a long time beyond birth. Similarly to null mice, their epiphyseal growth plates are drastically expanded.^28^ Treating adolescent rats with Gefitinib, an EGFR‐specific inhibitor, for a week reproduces this phenotype.^29^ Manipulation of EGF‐like ligands achieves the same effects. For example, either knockout of TGFα^30^ or its sheddase Adam17^31^ results in the accumulation of terminally differentiated chondrocytes in the growth plate, with delayed ossification. On the contrary, overexpressing BTC ubiquitously leads to a smaller hypertrophic cartilage zone regardless of age.^32^ Interestingly, although EGF and EGFR are expressed in all chondrocytes throughout the entire growth plate cartilage,^33^ only the hypertrophic zone but no other zones are affected in those animal models. These results suggest that although EGF‐like ligands are potent mitogens for chondrocytes in culture, they actually have a minimum effect on the proliferation and differentiation of growth plate chondrocytes in vivo. An alternative explanation is that inactivation of one ligand or one receptor does not result in a proliferation defect due to potential redundancy in the system.

Considering growth plate cartilage is constantly generated and degraded during endochondral ossification, these observations clearly indicated that a possible action of EGFR signaling in growth plate development is the stimulation of cartilage matrix degradation for cartilage remodeling into bone. At the chondral‐osseous junction (COJ) between growth plate and metaphyseal bone, osteoclasts and terminally differentiated chondrocytes are the major sources of matrix metalloproteinases (MMPs) that degrade cartilage matrix. Initially, study of surviving *Egfr* null mice revealed a decreased number of osteoclasts at the COJ.^26^ A similar phenotype was observed in mice deficient for *TGFα*.^30^ Because osteoclasts express *Egfr* gene and the EGFR inhibitor AG1478 completely abolishes osteoclastogenesis in vitro, it was concluded that EGFR regulates POC formation and growth plate development through defective osteoclast recruitment.^26^

However, later studies pointed out that in addition to the regulation of chondrocyte proliferation and differentiation, EGFR signaling also has catabolic action on chondrocytes by modulating their expression of MMPs and osteoclast regulatory factors (Fig. 2 *B*). MMP9, 13, and 14 are the major MMPs for growth plate cartilage degradation.^34^ Knockout of either of these MMPs causes an expanded growth plate cartilage,^35^ a similar phenotype as that observed in EGFR‐deficient mice. Their expression levels, particularly MMP13, are drastically decreased in the terminally differentiated chondrocytes at the COJ of growth plate in several rodent models with diminished EGFR activity, including EGFR inhibitor‐treated rats,^29^ *TGFα* knockout mice,^30^ and *Adam17* knockout mice.^36^ In vitro, TGFα increases both mRNA and protein amount of these MMPs in primary chondrocytes.^29^ In addition, the RANKL/OPG axis is the major route regulating osteoclastogenesis.^37^ Our studies have shown that TGFα is able to upregulate RANKL and downregulate OPG in chondrocytes, thus favoring bone resorption by osteoclasts.^29^ This is consistent with another report that the expression of RANKL and OPG is reciprocally regulated in a similar manner in *Adam17* knockout chondrocytes.^36^

Analyzing EGFR downstream signaling pathways reveals that upregulation of MMP9 and RANKL by EGFR signaling is partially mediated by the canonical Wnt/β‐catenin pathway, whereas EGFR‐enhanced MMP13 expression is not.^38^ Moreover, the elongation of growth plate is observed in cartilage‐specific *Egfr* knockout mice using either Col2‐Cre^29^ or Runx2‐Cre,^39^ as well as upon *Adam17* ablation using Col2‐Cre,^36^ but not in osteoclast‐specific *Egfr* knockout mice,^39^ proving that EGFR action on growth plate development is mainly mediated by a direct action on chondrocytes. Consistent with effects on POC and growth plate development, mice with deficient EGFR activity or TGFα null also exhibited delayed SOC formation,^30^, ^38^ further confirming the essential role of chondrogenic EGFR signaling in the cartilage‐to‐bone transition during skeletal development.

## EGFR Signaling in Articular Cartilage Development and Maintenance

As part of the knee, articular cartilage is a layer of chondrocytes covering the ends of long bones. Different from growth plate cartilage that plays a crucial but only transient role in the skeleton, articular cartilage is a permanent tissue acting throughout life. Its principal function is to provide a smooth, lubricated surface for low friction articulation, as well as load transmission and energy dissipative shock absorption during joint movement. The origin of articular cartilage is also different from growth plate cartilage. While growth plate chondrocytes derive directly from chondrocytes in the mesenchymal condensation, articular cartilage cells are derived from interzone cells that segment the mesenchymal condensation. In hindlimb development, mesenchymal condensation initially appears as a continuous Y‐shaped structure. Right before POC formation, cells at the future knee joint site increase their density and become flattened to form an interzone structure that later gives rise to articular cartilage, synovial lining, and other joint tissues.^40^ Therefore, the arms of Y‐shape structure eventually become tibia and fibula and the shaft becomes the femur. EGFR signaling does not appear to participate in this early stage of articular cartilage formation because no report of joint abnormality has been reported in any genetically modified mouse models of *Egfr* and its ligands. Of course, the redundancy in ligands and/or receptors might have prevented identification of such a role in current models.

At birth, the articular cartilage layer is isotrophic in structure and can be distinguished from the underneath epiphyseal cartilage by matrilin‐1 expression level.^41^ During postnatal development, while epiphyseal cartilage is soon replaced by bone to form the SOC through the endochondral ossification process, articular cartilage stays and its cells reorganize into a highly anisotropic structure with well‐defined vertical columns and horizontal layers. At the adult stage, mature articular cartilage is composed of four zones: superficial, transitional, middle, and calcified zones.^42^ The combination of the first three zones is also termed the uncalcified zone. Among them, the outmost superficial layer is different from other zones. It consists of two to four layers of flat cells expressing unique molecules, such as proteoglycan 4 (Prg4, lubricin), and contains a fine network of collagen fibrils that are oriented horizontally and parallel to the articular surface.^43^ It has multifaceted roles in maintaining proper joint functions. Those roles include, but are not limited to, producing lubricant proteins, harboring chondroprogenitors, resisting shear stresses, serving as a gliding surface, and isolating deeper layers from synovial fluid.^43^ However, the regulation of this special layer of articular cartilage is still largely unknown.

**Figure 3:**
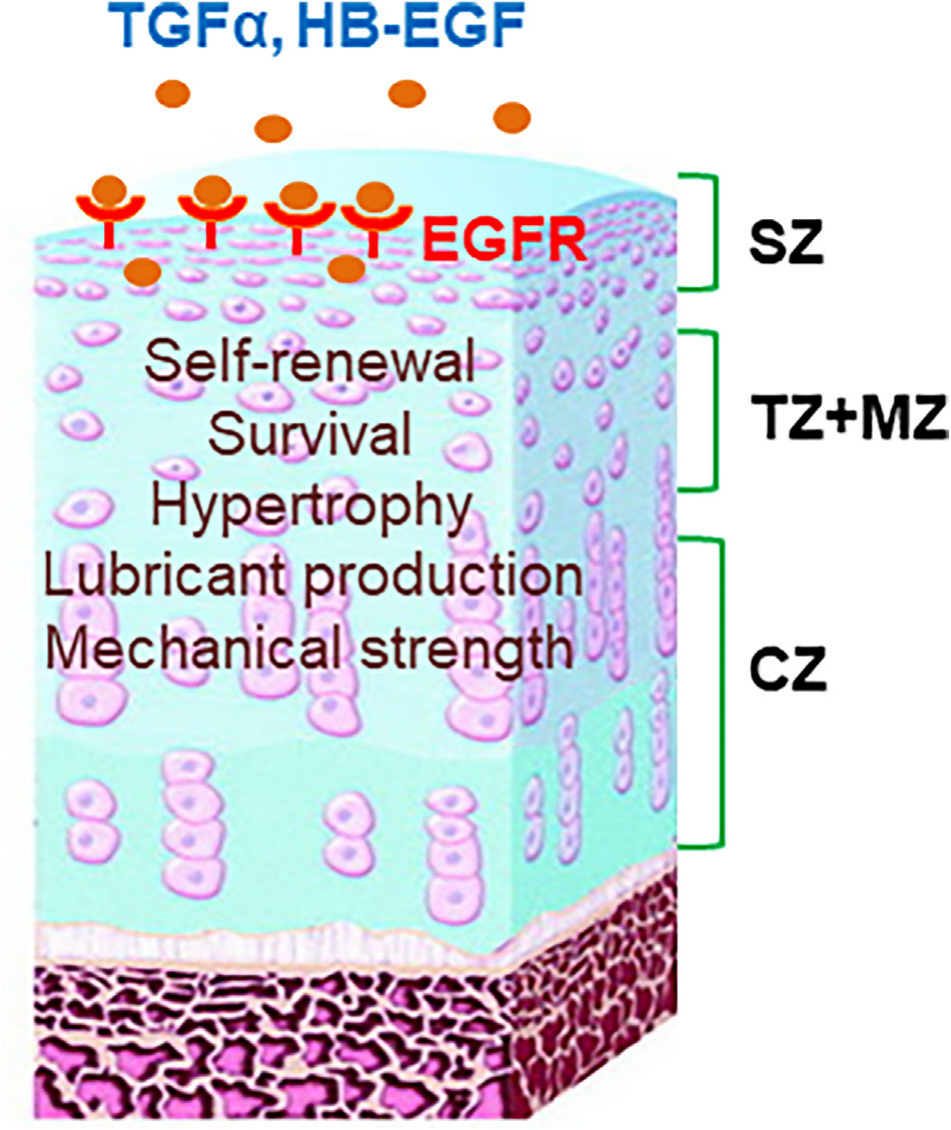
The role of EGFR signaling in articular cartilage development. Adult knee articular cartilage is composed of superficial zone (SZ), transitional zone (TZ), middle zone (MZ), and calcified zone (CZ). Activated EGFR is mainly detected in the top part of articular cartilage. TGFα and HB‐EGF are the most abundant EGFR ligands in the articular cartilage. Activation of EGFR signaling by its ligands is essential for promoting proliferation, survival, and lubrication production of superficial chondrocytes and for preventing their hypertrophy. EGFR signaling is also important for maintaining the mechanical strength of the cartilage surface.

To date, studies from our group and others demonstrated that EGFR signaling is critical in maintaining the number and function of postnatal articular cartilage chondrocytes, particularly those in the superficial layer (Fig. 3). EGFR, its partner ERBB2, and its various ligands are expressed in normal articular cartilage. Knockout of a single ligand, *TGFα*, does not appear to affect the structure and morphology of articular cartilage, suggesting redundancy of EGF‐like ligands in this tissue.^44^ Although EGFR staining is found in all four zones, the activated form of EGFR, p‐EGFR, and downstream signal p‐ERK are largely restricted to the distal part of articular cartilage, particularly the superficial zone.^45^, ^46^, ^47^, ^48^

Mitogen‐inducible gene‐6 (Mig6, or ErbB receptor feedback inhibitor 1, Errfi1) is an endogenous EGFR inhibitor that binds to an extended surface of the catalytic domain in ligand‐activated EGFR, locks it in an inactive configuration, and thus prevents signal generation.^49^ In addition, Mig6 drives EGFR into late endosomes and promotes lysosome‐mediated degradation of EGFR.^50^ Mig6 inhibits downstream EGFR signaling, including p‐ERK and p‐AKT, as well as biological responses regulated by EGFR, such as cell proliferation. Mig6 protein is mainly located in the superficial zone of normal mouse articular cartilage, a pattern similar to p‐EGFR. An early study using global ablation of *Mig6* in mice, which results in elevated EGFR activity, found enlargement and deformity of major joints.^51^ Later, cartilage‐specific (Col2‐Cre)^46^, ^48^ or skeletal‐specific (Prx1‐Cre)^47^ knockout of *Mig6* resulted in increased proliferation of superficial chondrocytes and therefore a thickened layer of adult articular cartilage. On the contrary, cartilage‐specific inactivation of EGFR in mice leads to fewer superficial chondrocytes at the age of 2 months.^45^ Moreover, these *Egfr* CKO mice display reduced proliferation, increased apoptosis, and chondrocyte hypertrophy in the top layer of articular cartilage. These in vivo phenotypes are consistent with in vitro culture data showing that EGFR signaling regulates proliferation, survival, and differentiation of superficial chondrocytes. Recent studies have implicated that the superficial layer harbors chondroprogenitors for articular cartilage.^52^, ^53^ Therefore, it appears that the role of EGFR signaling in articular cartilage is in line with its general function on tissue‐specific stem cells: maintaining the self‐renewal of stem cells and preventing their premature differentiation.

In addition, we have demonstrated that EGFR signaling promotes the lubrication function of the articular surface by increasing the amount of boundary lubricants, Prg4 and HA, from superficial chondrocytes.^45^ Tribological testing in bovine cartilage explant culture confirmed that EGFR signaling is an important regulator of cartilage lubrication function. Moreover, we detected remarkable decreases in the effective nanoindentation modulus in the surface of articular cartilage of *Egfr CKO* mice,^45^ suggesting that EGFR signaling also regulates mechanical properties of articular cartilage.

## The Importance of EGFR Signaling in Osteoarthritis (OA)

When reaching adult stage, chondrocytes in articular cartilage stop proliferating and enter a low turnover status of maintaining the cartilage extracellular matrix via their anabolic and catabolic actions. Aging, metabolic dysfunction, or mechanical injury can disrupt this balance and cause progressive cartilage degeneration. Unfortunately, articular cartilage has limited abilities to maintain and repair itself, and with age these abilities further decline. The degeneration of articular cartilage and associated changes in synovium and subchondral bone result in joint pain and dysfunction that characterize OA.

Interestingly, studying human OA cartilage samples identified two EGFR ligands, TGFα and HB‐EGF, to be expressed at a higher level than those in normal cartilage.^54^, ^55^ Furthermore, two genomewide association studies (GWAS) of tens of thousands of samples revealed TGFα as one of the genes most associated with the thickness of hip cartilage^56^ and human OA.^57^ Various surgical techniques have been developed to induce OA in adult rodent knee joints.^57^ In rats receiving anterior cruciate ligament (ACL) transection and partial medial meniscectomy combined with forced mobilization, TGFα protein amount appeared to be increased at the damaged cartilage surface.^54^ In mice receiving destabilization of the medial meniscus (DMM) surgery, the mRNA level of HB‐EGF increased in the knee joint at 2 months post‐surgery only in old mice (DMM at 12 months of age) but not in young adult mice (DMM at 12 weeks of age).^55^ Coincidently, our RNA analysis suggested that these two ligands are the most abundant EGFR ligands in healthy articular cartilage.^45^

Both TGFα and HB‐EGF bind and signal through EGFR in chondrocytes. During OA initiation, we found that EGFR activity in the top layer of articular cartilage is diminished in both human and mouse samples.^45^ Since early studies have demonstrated that OA initiation in dog and cyclic impact loading on cartilage explants significantly elevate Mig‐6 mRNA level in chondrocytes,^58^, ^59^ we reason that the downregulation of EGFR activity during early OA is potentially due to the abnormal loading‐induced Mig6 action and therefore could occur without changes in ligand availability. At the late stage of OA, it was reported that human OA samples (between 3 and 4 in the Kellgren and Lawrence grading system),^60^ as well as rat samples after OA surgery,^54^ contain significantly more activated EGFR than controls. These temporal changes of EGF‐like ligands and EGFR activity during OA progression strongly implicate that EGFR signaling is involved in OA pathology but possibly in a stage‐dependent manner.

To date, the consensus of most studies on EGFR signaling in OA is that like some other growth factor signaling pathways, EGFR exerts dual actions on articular cartilage. On the one hand, it plays an anabolic action by stimulating chondrocyte proliferation and survival. Because chondrocyte proliferation is rare in adult and aged articular cartilage, its survival action is likely the main contributing factor for maintenance of cartilage. In addition, EGFR signaling promotes the lubrication function of the articular surface.^45^ Since lubricants are essential for normal joint movement, EGFR functions to protect the superficial layer against insult at the OA initiation stage. On the other hand, EGFR signaling also plays a catabolic action by inhibiting the expression of the chondrogenic master transcription factor Sox9, thereby suppressing the synthesis of cartilage matrix proteins, such as type II collagen and aggrecan. On the other hand, EGFR signaling stimulates the expression of matrix degradation proteinases, such as MMP13.^25^, ^45^, ^54^, ^55^, ^60^, ^61^ Therefore, the overall effect of EGFR signaling on OA development is determined by the balance of these two opposite actions and most likely is age‐, OA stage‐, and context‐dependent.

## EGFR Signaling in Mouse Models With Aging‐Related OA

Genetically modified animal models targeting EGFR, its ligand, and its regulator Mig‐6 have been analyzed to understand the role of EGFR signaling in aging‐related OA. An early study of *Mig6* global knockout noticed cartilage degeneration in those mice at 4 months of age.^51^ Similar cartilage degeneration was also observed in mice with skeletal‐specific (Prx1‐Cre) Mig‐6 deletion at the same age, albeit the phenotype was much milder.^47^ However, mice with cartilage‐specific knockout of *Mig‐6* (Col2‐Cre) did not exhibit obvious abnormality at the articular cartilage surface at 36 weeks^46^ and 13 to 16 months of age,^48^ indicating that hyperactive EGFR signaling in chondrocytes does not cause cartilage matrix degradation. Interestingly, one common feature of all those *Mig6* knockout mouse models is ectopic cartilage formation and endochondral ossification in surrounding structures of the knee. It is important to keep in mind that Mig‐6 also fine‐tunes other receptor tyrosine kinases, such as HGF/MET.^62^ Therefore, interpretation of Mig‐6 mouse data has limitations.

On the other hand, articular cartilage phenotypes have been studied in aged mice with diminished EGFR signaling. We have reported spontaneous OA initiation in mice with cartilage‐specific (Col2‐Cre) *Egfr* knockout mice at 6 months of age. Another 6 months later, these mice displayed moderate OA phenotypes accompanied by subchondral bone sclerosis at both medial and lateral sites of articular cartilage, due to abnormal loading for a long period of time.^63^ These results are in line with the role of EGFR in articular cartilage development because those *CKO* mice have already developed reduced number of superficial chondrocytes, decreased lubricant secretion at the cartilage surface, and weakened mechanical properties at a young age.^45^ Meanwhile, deletion of one EGFR ligand, TGFα, globally did not affect cartilage morphology in knee, elbow, and ankle joints of 18‐month‐old mice.^44^ This could be explained by the redundancy of EGFR ligands and/or ligand‐independent actions of EGFR.

## EGFR Signaling in Mouse Models With Injury‐Induced OA

Owning to its important action in cancer development, the structure of EGFR protein has been extensively studied with a goal of designing specific and potent inhibitors. Currently, two small molecular inhibitors are used for clinical treatment of lung and breast cancers: gefitinib and erlotinib. Those inhibitors, along with others, target the ATP binding site of EGFR kinase domain and suppress its kinase activity.^64^ As shown in Table 1, {TBL 1} several rodent studies using those inhibitors have been performed to illustrate the role of EGFR signaling in injury‐induced OA development in mice and rats. Unfortunately, these studies do not achieve consent conclusions. Although all studies started inhibitor treatment right at or shortly after OA surgery, these studies differ in specific compounds used, dosage, administrative route, animal species, strain, sex, and surgery chosen. Because these inhibitors might also have off‐target effects, it is difficult to interpret the outcomes solely based on EGFR actions.

**Table 1:** A List of Reports Studying the Effect of EGFR Inhibitors on Mouse Knee OA Progression

EGFR inhibitor	Animal	Surgery model	Treatment	OA progression	Reference
Gefitinib	Male 3‐month‐old 129S2 mice	DMM	Oral gavage at 100 mg/kg once every other day for 12 weeks	Accelerated OA at 12 weeks post‐surgery	67
AG1478	Male 11‐ to 12‐week‐old Sprague‐Dawley rats	ACL transection and partial medial meniscectomy	Continuous infusion at 6.6 µg/kg/hr for 4 to 10 weeks	Reduced OA at 4 and 7 weeks but no change in OA severity at 10 weeks post‐surgery	68
Erlotinib	Male and female 13‐week‐old BALB/c mice	DMM	Oral gavage at 50 mg/kg/d for 12 weeks	Reduced OA at 12 weeks in females post‐surgery; no change in OA severity or even worsened OA in males	69
Gefitinib	Male 2‐month‐old C57BL/6 mice	DMM	Oral gavage at 25 mg/kg/d for 8 weeks or intra‐articular injection of chitosan microspheres with gefitinib once every 3 days for 8 weeks	Reduced OA at 8 weeks post‐surgery in both types of treatment	60

OA = osteoarthritis; DMM = destabilization of the medial meniscus; ACL = anterior cruciate ligament.

Genetically modified mouse models also generated inconsistent and sometimes conflicting results. OA surgery (transection of the cruciate and meniscotibial ligaments, and meniscus removal) on 4‐week‐old *Mig‐6* global knockout mice resulted in a significant increase of OA histologic score at 1 month after surgery. However, this increase is mild and possibly not due to the surgery when considering the fact that those mice have already initiated spontaneous OA at this age.^65^ Knocking down *TGFα* protects young (10‐week‐old) but not adult (6‐month‐old) mice from DMM‐induced OA. But even in young mice, the protection effect is more prominent at 7 weeks than later at 14 weeks post‐surgery.^44^ In our studies, DMM surgery in 3‐month‐old cartilage‐specific *Egfr CKO* mice causes the most severe OA phenotypes at 2 to 3 months post‐surgery, including a complete loss of cartilage, extremely high surface modulus, subchondral bone plate thickening, and elevated joint pain.^45^ Interestingly, this effect is dependent on the dosage of EGFR activity. To generate *Egfr CKO* mice, we introduced a dominant negative allele of *Egfr, Wa5*,^66^ to achieve the maximum inactivation of EGFR activity. As a control, *Egfr^Wa^* ^5*/flox*^ mice always showed higher OA severity than wild‐type (WT) mice but much less than *Egfr CKO* mice after surgery.^45^ Using the severe OA model of *Egfr CKO*, we established mechanical loading‐induced attenuation of Sclerostin and elevation of bone formation along the SBP surface as the major mechanism for subchondral bone phenotypes associated with late‐stage OA in mice.^63^ However, this noninducible mouse model does not distinguish whether the severe OA phenotypes after DMM are due to the inferior nature of cartilage before the surgery or the requirement of EGFR signaling in response to acute insults. In contrast, a recent study found that inducible KO of *Egfr* in cartilage using Col2‐CreER, a system used more frequently in young mice rather than adult mice, and tamoxifen application right before DMM surgery results in protection of cartilage health compared with WT mice.^60^ Thus, similar to the pharmacological approaches discussed above, different studies on genetic inactivation of different components of the EGFR signaling system or even the same component with different approaches come to opposing conclusions. Nevertheless, these results highlight the importance and complexity of EGFR signaling in OA disease and beg for extensive future studies to delineate the exact role of this pathway in OA.

## Conclusions

In summary, decades of studies have concluded that EGFR signaling plays an essential role in cartilage development and homeostasis and therefore presents a novel target for treating cartilage diseases. Available data agree on an important role of this pathway in the transition from cartilage to bone during endochondral ossification. Similarly, all existing data support an anabolic role of EGFR signaling in articular cartilage formation and growth. However, the effects of EGFR inhibition on rodent models of OA are far less clear and appear often contradictory, hindering further translational studies on targeting this pathway for OA treatment. Therefore, we propose that the actions of EGFR pathway in OA progression are context‐specific. Multiple factors, such as the subtype of OA, OA stage, age, sex, other biological variables, specific ligand‐receptor combinations involved, and even housing conditions (including potentially the microbiome) could affect the final experimental outcomes. Future extensive studies on human cartilage samples and more mouse models are required to address all of these parameters, pinpoint the role of each ligand and receptor, and determine downstream signaling pathways. Such comprehensive studies will allow us to fully elucidate the role of EGFR pathways in OA and their suitability as therapeutic targets.

## Disclosures

The authors state that there is no financial support or other benefits from commercial sources for the work reported on in this article and they have no financial interests that could create a potential conflict of interest or the appearance of a conflict of interest with regard to the work.
